# The Role of Allopregnanolone in Pregnancy in Predicting Postpartum Anxiety Symptoms

**DOI:** 10.3389/fpsyg.2019.01033

**Published:** 2019-07-16

**Authors:** Lauren M. Osborne, Joshua F. Betz, Gayane Yenokyan, Lindsay R. Standeven, Jennifer L. Payne

**Affiliations:** ^1^Department of Psychiatry and Behavioral Sciences, Women’s Mood Disorders Center, Johns Hopkins University School of Medicine, Baltimore, MD, United States; ^2^Department of Gynecology and Obstetrics, Johns Hopkins University School of Medicine, Baltimore, MD, United States; ^3^Department of Biostatistics, Johns Hopkins Bloomberg School of Public Health, Baltimore, MD, United States

**Keywords:** pregnancy, postpartum, allopregnanolone, hormones, depression, anxiety

## Abstract

Postpartum depression is a serious illness affecting up to 15% of women worldwide after childbirth, and our understanding of its biology is limited. Postpartum anxiety is perhaps more prevalent and less understood. Prior studies indicate that allopregnanolone, a metabolite of progesterone, may play a role in reproductive mood disorders, including postpartum depression, but the exact nature of that role is unclear. Our own prior study in a group of psychiatrically ill women found that low allopregnanolone in the second trimester predicted the development of postpartum depression. In the present study, in both healthy and mood- and anxiety-disordered women who remained well throughout the perinatal period, we found that second trimester allopregnanolone predicted postpartum anxiety symptoms, with a similar trend toward the prediction of postpartum depressive symptoms (though without statistical significance). Both concurrent sleep and prior histories of mood and anxiety disorders contributed to the variance in mood and anxiety scores at 6 weeks postpartum. These findings confirm the importance of pregnancy allopregnanolone in postpartum psychiatric symptoms and point to future directions that may determine other important contributing factors.

## Introduction

Postpartum depression (PPD) is a serious illness affecting up to 15% of women worldwide after childbirth (Yonkers et al., 2011), with higher rates in populations with significant psychosocial stressors. The most serious consequence of PPD, suicide, is a leading cause of maternal death in the first year postpartum worldwide (Shadigian and Bauer, 2005; Storm et al., 2014; Iacobucci, 2016; Khalifeh et al., 2016; Metz et al., 2016). Less devastating but nonetheless serious effects include poor mother-infant bonding and effects on both cognitive and emotional development in the child (Stein et al., 2014; Netsi et al., 2018). Postpartum anxiety is equally, if not more, prevalent, and is less studied and understood (Dennis et al., 2017). The timing of symptoms in vulnerable women is coincident with the abrupt withdrawal from pregnancy levels of estrogen and progesterone at parturition (Halbreich and Kahn, 2001; Wisner et al., 2015)—but just what makes these women vulnerable is still unknown. Most studies have not found a relationship between absolute levels of hormones or the degree of decrease in levels (Heidrich et al., 1994; Harris et al., 1996; Chatzicharalampous et al., 2011; Schiller et al., 2015; Yim et al., 2015) and the development of PPD, indicating that individual women’s vulnerability to the change in hormone levels is likely more important than differences in absolute levels.

Recently, there has been considerable interest in the role of allopregnanolone (ALLO), a 3α-reduced metabolite of progesterone that is a potent allosteric modulator of the GABA-A receptor and may be responsible for the neuroprotective, anxiolytic, and sedative properties of progesterone (Schule et al., 2014). Some studies (Hellgren et al., 2014; Crowley et al., 2016) have found associations between lower levels of ALLO and mood in the perinatal period, but others have not found a relationship (Deligiannidis et al., 2013). Confusingly, this is opposite to the relationship found in premenstrual dysphoric disorder (PMDD), where numerous studies have found that elevated levels of ALLO in the luteal phase are associated with increased mood symptoms (Girdler et al., 2001; Martinez et al., 2016; Timby et al., 2016), leading some to suggest that mood and anxiety responses to ALLO may follow an inverted U-shaped curve, with both low and high levels being anxiogenic and a “sweet spot” in the middle being anxiolytic (Backstrom et al., 2014).

Our group has sought to examine the relationship between mood and ALLO across the perinatal period. In a prior study, we showed that lower levels of ALLO at the second trimester of pregnancy (T2) predicted the development of a postpartum depression, with each additional ng/ml of ALLO reducing the odds of PPD by 62% (95% *CI* = 13–84%, *p* = 0.022) (Osborne et al., 2017). That study was conducted in a population of psychiatrically ill women, all of whom had a history of a mood disorder, most of whom remained on antidepressants throughout the study, and half of whom developed PPD. We were not certain whether our results would be generalizable to a less ill population and therefore sought to examine a similar question [whether T2 ALLO can predict depressive or anxious symptoms at 6 weeks postpartum (W6)] in a different population – one that is roughly equally divided among women with and without histories of mood and/or anxiety disorders, with almost all women (regardless of history) remaining psychiatrically well throughout the perinatal period.

## Materials and Methods

### General Study Procedures

This was a prospective study conducted at The Johns Hopkins University School of Medicine in Baltimore, Maryland, USA. The study was approved by the Institutional Review Board of The Johns Hopkins University. We recruited both women with preexisting mood and anxiety disorders and healthy controls. Prior history of mood or anxiety disorder was determined by a thorough psychiatric interview conducted by an experienced perinatal psychiatrist, using DSM-IV criteria. Participants (*N* = 124) could enroll at any point in pregnancy, were seen for study visits at each trimester and at 6 weeks postpartum, and were managed clinically by their treating psychiatrists. Data collection included the Edinburgh Postnatal Depression Scale (EPDS) (Cox et al., 1987) for depressive symptoms and both the Spielberg State-Trait Anxiety Inventory (STAI) (Version Y) (Ramanaiah et al., 1983) and the Perinatal Anxiety Screening Scale (PASS) (Somerville et al., 2014) for anxiety symptoms, measures of stress, baseline clinical diagnoses by the Structured Clinical Interview for DSM-IV diagnoses (administered by a trained research assistant), as well as by psychiatrist interview, personality measures, sleep quality measures [Pittsburgh Sleep Quality Index (PSQI)] (Buysse et al., 1989), medication use, and a blood draw for biological factors.

### Hormone Analysis

Participant blood was collected at each visit in four 10-ml EDTA tubes. Blood samples were nonfasting, and collection times were arranged at the convenience of the participant. All occurred during the working day (9:00 a.m. to 5:00 p.m.). Samples were immediately centrifuged at 4°C for 30 min. The plasma was then aliquotted in 2 ml microcentrifuge tubes, snap frozen on dry ice, and immediately stored in a −80°C freezer. Blood was analyzed with the allopregnanolone EIA kit from Arbor Assays LLC Cat 3 KC44-H1. All samples were run in duplicate, and the coefficient of variation (CV) among samples was <10%. Predicted hormone concentrations were reshaped and merged with participant IDs prior to analysis.

### Statistical Analysis

Participants were included in the analytic cohort if they had both a blood sample at T2 and completed psychological scales at W6. Twenty-four subjects (19.4%) enrolled in the study after T2 and so did not have a blood draw at T2. Seven participants (5.6%) did not complete any psychological scales at W6, and one participant (0.8%) was missing covariates, leaving a total of 92 participants in the analytic cohort. Negative Binomial Generalized Linear Models (GLMs) were used to explore relationships between the log of ALLO in the second trimester and week 6 postpartum psychiatric outcomes (as measured by the EPDS, STATE score of the STAI, and PASS). Models were adjusted for maternal age, sleep (PSQI Global Score), and timing of week 6 postpartum visit. The relationship with ALLO was additionally explored by history of mood disorder, history of anxiety disorder, and history of psychiatric medication usage in three separate models. Each model included a main effect of the variable, as well as an interaction between the variable and log ALLO. Model assumptions were assessed using residual diagnostic plots. All continuous covariates (maternal age, visit timing, PSQI) were centered at their respective sample means. Robust standard errors were used in hypothesis tests and to construct 95% confidence intervals. Statistical analyses were performed using R version 3.5.1^1^.

## Results

Ninety-two women met criteria for inclusion in these analyses. Of those women excluded from analysis, 65.6% were white (compared to 89.1% of those included, *p* = 0.0049) and 78.1% were married (compared to 94% of those included, *p* = 0.013); there were no other significant differences between groups. The average age of the women included was 32.5 (*SD* = 3.7), and they were highly educated (with 61.5% having attained a graduate or professional degree); 21.4% of those with available medication history used psychiatric medications during the index pregnancy. The majority (58.7%) had a mood disorder, and 35.9% had an anxiety disorder. Mood disorder diagnoses broke down as 3.3% with bipolar I disorder; 3.3% with bipolar II disorder; and 47.8% with major depressive disorder. Complete demographic features of the entire sample, as well as those included and excluded in the analysis, are reported in Table 1. Most women, including those with mood disorders, remained psychiatrically well throughout the study. The median EPDS score at T2 was 4 (IQR 2, 7), at T3 4 (IQR 1, 7), and at W6 3 (IQR 1, 7), with few women attaining a score above the clinically meaningful cutoff of ≥13, indicating possible depression (5 at T2, 5 at T3, and 4 at W6). Median STAI State scores were 29 (IQR 23.8, 35) at T2, 29 (IQR 23, 37) at T3, and 27 (IQR 22, 39) at W6. Median PASS scores were 10 (IQR 5, 18.2), 10 (IQR 4.5, 18), and 10 (IQR 4.8, 18). There were more women with anxiety scores above a clinically meaningful cutoff (>35 on the STAI and ≥21 on the PASS), with 25 at T2, 27 at T3, and 30 at W6 for STAI and 16 at T2, 13 at T3, and 19 at W6 for the PASS. Mean log of ALLO levels were 1.6 at T2 (*SD* = 0.5), 2.2 at T3 (*SD* = 0.6), and −0.5 at W6 (*SD* = 0.6).

**Table 1:** Demographic characteristics of participants.

Variable	All (*N* = 124)	Included (*N* = 92)	Excluded (*N* = 32)
Mean age (*SD*)	32.6 (3.7)	32.5 (3.7)	32.9 (3.8)
Race			
White	103 (83.1%)	82 (89.1%)	21 (65.6%)
Black	11 (8.9%)	5 (5.4%)	6 (18.8%)
Asian/Pacific Islander	7 (5.6%)	3 (3.3%)	4 (12.5%)
Relationship status			
Single	5 (4%)	2 (2.2%)	3 (9.4%)
Married	112 (90.3%)	87 (94%)	25 (78.1%)
Widowed	0 (0%)	0 (0%)	0 (0%)
Cohabiting	6 (4.8%)	3 (3.3%)	3 (9.4%)
Education			
High school graduate	4 (3.3%)	2 (2.2%)	2 (6.2%)
Some college	7 (5.7%)	5 (5.5%)	3 (6.2%)
Bachelor’s degree	28 (22.8%)	22 (24.2%)	6 (18.8%)
Some graduate	8 (6.5%)	6 (6.6%)	2 (6.3%)
Graduate degree	76 (61.8%)	56 (61.5%)	20 (62.5%)
History mood disorder	76 (61.3%)	54 (58.7%)	22 (68.8%)
History anxiety disorder	47 (37.9%)	33 (35.9%)	14 (43.8%)
Taking psychiatric meds	24 (21.8%)	18 (21.4%)	6 (23.1%)
T2 ALLO (*SD*), ng/ml	5.2 (2.7)	5.3 (2.7)	4 (1.1)
Log T2 ALLO (*SD*)	1.5 (0.5)	1.6 (0.5)	1.4 (0.3)
W6 EPDS (IQR)	4 (1, 8)	3 (1, 5.8)	4 (2, 9)
W6 STATE (IQR)	27 (21, 31.5)	27 (22, 39)	25.5 (21, 33.8)
W6 PASS (IQR)	4 (2, 6)	4 (2, 6)	5 (2.5, 6)

*T2 = second trimester; ALLO = allopregnanolone; W6 = 6 weeks postpartum; EPDS = Edinburgh Postnatal Depression Scale; STATE = State score of the Spielberg State – Trait Anxiety Inventory, Version Y Totals do not equal 100% due to missing data*.

**Figure 1:**
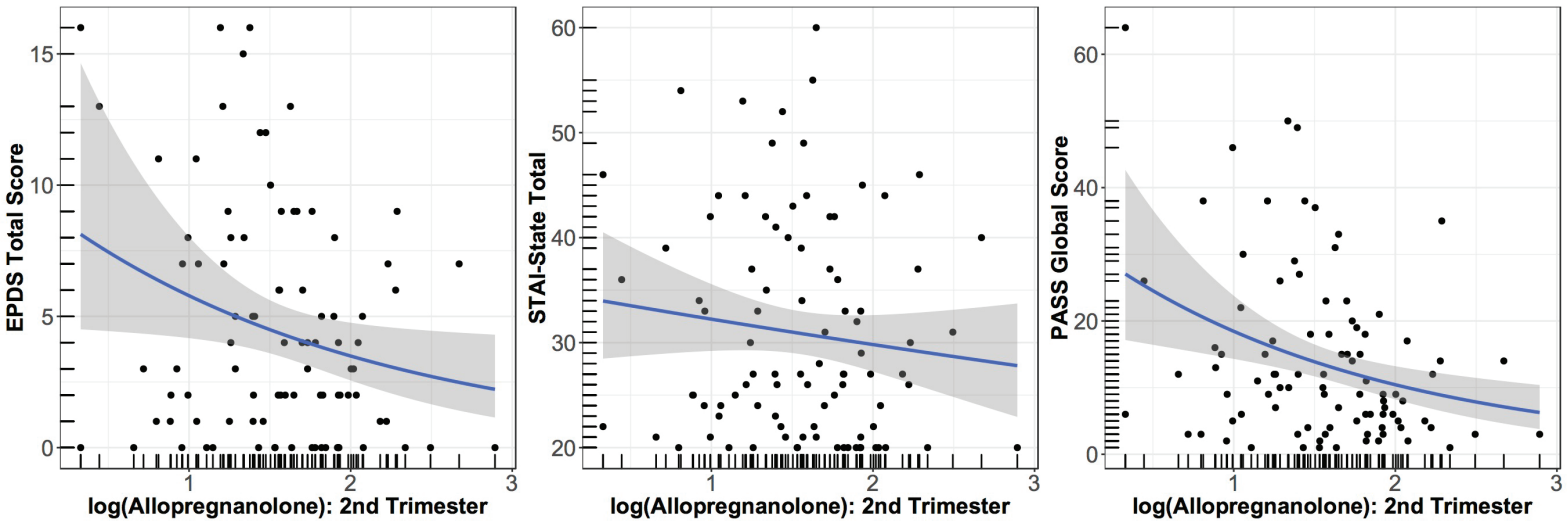
Relationship between EPDS score at W6 and log of ALLO concentration at T2 (*p* = 0.10); between STATE score at W6 and log of ALLO concentration at T2 (*p* = 0.55); and between PASS score at W6 and log of ALLO concentration at T2 (*p* = 0.025). Smoothed averages are shown from a negative binomial generalized additive model, with 95% confidence interval.

As seen in Figure 1, both EPDS and PASS scores in week 6 exhibited a negative association with log ALLO in the second trimester, with lower log ALLO at T2 being associated with higher symptom scores at W6; STAI scores exhibited a flatter trend. After adjusting for maternal age, gestational age, and sleep quality, higher log ALLO in the 2nd trimester was associated with lower EPDS and PASS scores (~30% lower scores per 1 unit increase in log ALLO in both outcomes), but STAI-State scores did not exhibit an association. Only the aggregate association between PASS and log ALLO was statistically significant at the 5% level (exponentiated *β* = 0.68, 95% *CI* = 0.48–0.97, *p* = 0.025). Concurrent sleep quality also accounted for some of the variation in W6 scores, with higher PSQI scores, indicating poorer sleep quality, related to higher symptoms on all inventories. All other things being equal, each unit increase in global PSQI score at W6 was associated with a 10% increase in the concurrent EPDS score (exponentiated *β* = 1.10, 95% *CI* = 1.02–1.17, *p* = 0.005), a nonsignificant 3% increase in the concurrent STATE score (exponentiated *β* = 1.03, 95% *CI* = 0.99–1.06, *p* = 0.073), and an 14% increase in the concurrent PASS score (exponentiated *β* = 1.14, 95% *CI* = 1.08–1.21, *p* < 0.001).

We then allowed relationships with ALLO in the regression models to vary by history of mood disorder, history of anxiety disorder, and concurrent medication use. In the adjusted model with an interaction, a history of mood disorder was associated with a 2.4-fold increase in the EPDS score at W6 compared to no history of mood disorder at the average level of log ALLO (estimated means at the reference level of all variables: no history of mood disorder = 1.99, 95% *CI* = 1.12–3.54; with history of mood disorder = 4.80, 95% *CI* = 3.81–6.06; exponentiated *β* = 2.41, 95% *CI* = 1.35–4.30, *p* = 0.003) and a 1.8-fold increase in the PASS score at W6 (estimated means at the reference level of all variables: no history of mood disorder = 8.06, 95% *CI* = 4.52–14.35; with history of mood disorder = 14.53, 95% *CI* = 11.51–18.33; exponentiated *β* = 1.80, 95% *CI* = 1.01–3.21, *p* = 0.045). The interaction term that included history of mood disorder and T2 log ALLO was statistically significant in the model for EPDS score (exponentiated *β* = 0.36, 95% *CI* = 0.14–0.90, *p* = 0.018), indicating opposite trends for the relationship of T2 ALLO to W6 scores depending on history of mood disorder diagnosis, although these trends did not reach statistical significance at the 5% level. Those who had no history of a mood disorder had a 90% increase in EPDS scores (exponentiated *β* = 1.94, 95% *CI* = 0.96–3.90, *p* = 0.065) for each log unit increase in T2 ALLO, while those who did have a history of a mood disorder had a 31% decrease in EPDS score (exponentiated *β* = 0.69, 95% *CI* = 0.43 to 1.10, *p* = 0.122) for each log unit increase in T2 ALLO. History of anxiety disorder did not affect the directionality of these relationships. In the adjusted model with interaction, history of anxiety disorder at the average level of T2 log-ALLO was associated with a 1.9-fold increase in EPDS at W6 (exponentiated *β* = 1.90, 95% *CI*, 1.33–2.73, *p* = 0.001) and a 1.7-fold increase in the PASS score at W6 (exponentiated *β* = 1.73, 95% *CI* = 1.21–2.49, *p* = 0.003). Concurrent medication use was not a significant effect modifier and did not have a statistically significant effect on W6 scores at the average level of T2 log-ALLO.

## Discussion

In a population of women with and without mood and anxiety disorders, almost all of whom remained psychiatrically well throughout the study, we found an association between lower T2 ALLO and higher anxiety scores at 6 weeks postpartum, as well as a similar relationship between T2 ALLO and higher W6 depressive symptoms, which did not reach statistical significance in the adjusted model. While this does not exactly replicate our previous findings, the intriguing additional finding of an opposite relationship between T2 ALLO and W6 mood scores depending on prior history of mood disorder may indicate that women with mood disorders may respond differently to allopregnanolone than those without. We also found that, controlling for the level of ALLO at T2, each additional increment of disrupted sleep at W6 (as measured by a one-point increase in the global PSQI score) was associated with an additional 10% increase in depressive symptoms and 11% increase in anxiety symptoms, and that women with histories of prior mood or anxiety disorders had a roughly two-fold increase in both EPDS and PASS scores in the postpartum compared to those with no history at the average level of T2 ALLO (in keeping with the amount that prior literature has found to represent a clinically important increase) (Matthey, 2004). Interestingly, our findings depended upon the tool used to measure anxiety. The relationship between pregnancy allopregnanolone and postpartum anxiety was detectable only with an instrument designed specifically for the perinatal population, the PASS; we did not detect a relationship when anxiety was measured with an instrument designed for the general population (the STAI).

Several factors may underlie the differences between this and our prior study. The prior study included measures of clinician-diagnosed depression, whereas this study used EPDS scores as a proxy for postpartum depressive symptoms; the two may not be comparable, and the EPDS, while well validated as a screening tool, is not a diagnostic tool (Cox, 2017). In the current population, at T2, only 5.4% had EPDS scores ≥13, indicative of possible depression, and only 4.3% were above that cutoff at 6 weeks postpartum. By contrast, in our prior study, 38% were depressed (by clinician diagnosis using DSM-IV criteria) at T2 and 48% at W6. As an additional indication of severity of illness, 21.4% of the subjects in the current study were using psychiatric medications, while 74% of the population in the prior study was on medications.

While T2 ALLO was not statistically significantly associated with W6 EPDS score, the point estimate and shape of the relationship were identical to those found in our prior study. In addition, the difference we found between women with and without a history of mood disorder (with those with history showing higher W6 EPDS scores for lower T2 ALLO, and those without history showing the opposite) may indicate that this relationship holds only for women with mood disorders. In our prior study, we were not able to assess anxiety symptoms; the association we found here between T2 ALLO and W6 anxiety symptoms may indicate that ALLO’s predictive value for PPD exists because of its effects on anxiety symptoms (as anxiety is a major clinical feature of PPD). In addition, we have shown a substantial effect of sleep, perhaps indicating that the path to anxiety and depression from ALLO may lie through poor sleep (or, conversely, that those with poor sleep may have lower levels of ALLO).

This is an exploratory study, and as such, there are substantial limitations. The sample size is small (though larger than that in our previous study), and most participants were highly educated white women. We were unable to control for some clinical confounders that could have affected our results, including body mass index, levels of other hormones, and other medical conditions. Blood was not collected at the same time point during the day for each subject, and it is possible that our results were affected by diurnal variations. We did not collect information about fetal sex and so were unable to examine any differences in mood, anxiety, or hormone level by sex of the fetus.

These results nevertheless indicate that allopregnanolone early in pregnancy continues to be an intriguing player in postpartum mood and anxiety symptoms; further studies on exactly how that relationship may work (what are the additional steps in the chain between second trimester ALLO and postpartum symptoms?) will prove a rich area of research. In addition, our work shows that sleep and especially prior history are also substantial independent factors. This should be good news for our field, as sleep interventions and careful screening for prior depressive episodes and/or anxiety disorders are low-cost tools that should be easy to implement and could make substantial improvements in our ability to prevent postpartum depression.

## Ethics Statement

This study was carried out in accordance with the recommendations of the Institutional Review Board of the Johns Hopkins University with written informed consent from all subjects. All subjects gave written informed consent in accordance with the Declaration of Helsinki. The protocol was approved by the Institutional Review Board of the Johns Hopkins University.

## Author Contributions

LMO collected data and wrote the paper. JLP and LRS collected data and edited the paper. JFB and GY designed and carried out statistical analyses.

### Conflict of Interest Statement

JLP receives research support from Sage Therapeutics and holds a patent for epigenetic biomarkers of postpartum depression.

The remaining authors declare that the research was conducted in the absence of any commercial or financial relationships that could be construed as a potential conflict of interest.

## Footnotes

^1^https://www.R-project.org
